# Compositional Analyses Reveal Relationships among Components of Blue Maize Grains

**DOI:** 10.3390/plants9121775

**Published:** 2020-12-14

**Authors:** Amol N. Nankar, M Paul Scott, Richard C. Pratt

**Affiliations:** 1 Department of Plant and Environmental Sciences, New Mexico State University, Las Cruces, NM 88003-8003, USA; ricpratt@nmsu.edu; 2 Center of Plant Systems Biology and Biotechnology (CPSBB), 4000 Plovdiv, Bulgaria; 3 USDA-ARS, Corn Insects and Crop Genetics Research Unit, Ames, IA 50011, USA; paul.scott@usda.gov

**Keywords:** blue maize, NIR calibration, NIR predictions, heirloom pigmented maize, grain compositional traits, chemometric models, reference analysis

## Abstract

One aim of this experiment was to develop NIR calibrations for 20-grain components in 143 pigmented maize samples evaluated in four locations across New Mexico during 2013 and 2014. Based on reference analysis, prediction models were developed using principal component regression (PCR) and partial least squares (PLS). The predictive ability of calibrations was generally low, with the calibrations for methionine and glycine performing best by PCR and PLS. The second aim was to explore the relationships among grain constituents. In PCA, the first three PCs explained 49.62, 22.20, and 6.92% of the total variance and tend to align with nitrogen-containing compounds (amino acids), carbon-rich compounds (starch, anthocyanin, fiber, and fat), and sulfur-containing compounds (cysteine and methionine), respectively. Correlations among traits were identified, and these relationships were illustrated by a correlation network. Some relationships among components were driven by common synthetic origins, for example, among amino acids derived from pyruvate. Similarly, anthocyanins, crude fat, and fatty acids all share malonyl CoA in their biosynthetic pathways and were correlated. In contrast, crude fiber and starch have similar biosynthetic origins but were negatively correlated, and this may have been due to their different functional roles in structure and energy storage, respectively.

## 1. Introduction

For a little over five decades, near-infrared reflectance (NIR) and transmittance (NIT) spectroscopy have been used in agriculture [1,2,3], animal sciences [4], and the pharmaceutical industry [5]. NIR has been used in the prediction of compositional traits across different cereals [6,7,8], soybeans [9], and red grapes [10]. The characteristic non-destructive sample preparation [11], reproducibility [12], ability to develop calibrations for specific components [13], and high prediction performance [14] make it an efficient method for rapid phenotypic screening inbreeding and other applications. Near-infrared spectroscopy is an inexpensive alternative to conventional analytical methods [15,16]. It can be used to screen samples either as ground flour using NIR [17] or as bulk whole grains by NIT [18]. The latter can allow the preservation of seeds that are limited in quantity or allow additional analyses of a batch of seeds [19,20]. In addition, single-seed NIRS facilitates the screening of samples from segregating populations [21], improved the efficiency of selection [22] and thereby increased genetic gain.

Maize displays extensive genetic diversity for grain physical and compositional traits. This variation lends itself to diverse end-uses-including food, feed, fiber, and industrial applications [23,24,25]. Maize is mainly used as a feed; however, it remains an important food source in many areas [14], making quantification of grain composition in regard to human nutrition desirable. In maize, NIRS has been utilized to evaluate several grain compositional traits [20,26,27,28], dry matter [29], physical attributes such as kernel hardness [30], mycotoxin levels [31,32], and phenolic content [13]. NIRS can be used for the selection of grain quality traits, but the diversity of maize grain phenotypes make a universal calibration extremely difficult to create.

The concentration, distribution, and association of maize grain biochemical constituents determine the grain quality. Most studies of maize grain quality have focused on the major components: starch, protein, oil, and nutritionally limiting essential amino acids such as cysteine, methionine, and lysine [28,33,34]. Analysis of non-essential amino acids, fiber, ash, and anthocyanin components is less common than for other grain constituents [33,35,36]. Examinations of correlations among different grain constituents are limited and detailed understanding would be of interest to maize breeders, geneticists, biochemists, and food scientists. Starch has been widely reported as being negatively correlated with oil and protein, whereas positive correlations between oil and protein [28,33] have been observed. Most amino acids have been reported to be positively correlated with protein and negatively correlated with starch [33]. The only noticeable positive correlation among amino acids has been reported between the essential amino acids lysine and tryptophan [35,37].

To date, most NIR studies have been limited to yellow flint, dent and pop maize and NIRS calibrations were reported primarily for major constituents. NIR applications and calibration development in blue maize have lagged. Calibrations based on yellow maize samples may not predict blue maize composition well. Interest in blue maize is rising as demand for specialty and ethnic food products continues to increase [28,36]. In this study, we aimed to develop NIRS calibration for compositional traits in anthocyanin pigmented maize grain. We sought to (a) develop whole-grain calibration for blue, red and purple maize compositional constituents, (b) validate developed calibrations to determine calibration quality, and (c) characterize the relationship among different grain constituents.

## 2. Results

### 2.1. Descriptive Statistics and Trait Variation

The variability of wet lab analyzed grain composition in blue, purple, and red grain maize samples were examined in developing the NIR calibrations. Anthocyanins, total fatty acids, and oil content had greater variation compared to other traits, while starch displayed the least variation. Among amino acids, proline, methionine and leucine showed the largest variation and lysine showed the least (Table 1). The ANOVA across accessions, locations and years was also tested (Table 2), and accessions had significant differences for total fatty acids, oil, crude fiber, ash, anthocyanin, methionine, and lysine; however, accessions were not significantly different for starch, protein, essential (threonine, valine, isoleucine, and leucine) and conditionally essential amino acids (proline and glycine). Fixed effects of location and year showed significant differences for all traits except for total fatty acids and oil (Table 2). Interactions between accession and location did not show any significant differences except for anthocyanin, whereas the interaction between accession and year were also nonsignificant for all traits except aspartic acid, threonine and anthocyanin. Highly significant *F*-values to test the variation among accessions suggest that variability between replicates within accession was relatively small by comparison.

Standard errors of NIRS predicted values for protein, lysine, and methionine were higher than those of the reference analysis, while standard errors of NIRS predicted values for oil, starch, and cysteine were lower in comparison to reference analysis (Table 3). RMSEP is a measure of prediction performance estimated from predictions of a subset of samples that are not in the calibration set. Standard errors of calibration (RMSEP) of protein, oil, starch, cysteine, methionine and cysteine were around ten times for PCR and PLS than those of laboratory errors of the reference analysis (Table 3).

### 2.2. Performance Statistics of Calibration and Validation Sets

Calibration equations for each analyzed component were built with different spectral combinations and mathematical processing methods, and the calibration equation for the 0th and first derivatives is shown in Table S2A and S2B, respectively. A variety of derivatives were tested for PCR and PLS models and they had little effect on the performance of prediction equations among the compositional traits. Evaluation of the predictive ability of the calibration models was performed by validation with an independent, randomly selected subset of the data. Summaries of validation statistics for measured grain constituents using 0th and first derivatives are shown in Table 4 and Table 5, respectively and details about each calibration parameter estimated by calibration models for 0th and first derivative are shown in Table S1A and S1B, respectively. When comparing the R^2^, RMSEC, RMSEP, and RPD values, it is clearly seen that the two derivatives tested gave similar prediction ability of calibration equations. Results showed the highest correlation between validation and reference data sets was 0.36 (Table 4 and Table 5) for both derivatives. RMSEP values were low, suggesting that the validation sets represented the whole data set well. RPD, a measure of the standard error of prediction relative to the standard error of the reference chemistry were above 0.8 in most cases, suggesting the predictive value of the calibration models is good.

### 2.3. Multivariate Analysis

#### 2.3.1. Principal Component Analysis

**Figure 1:**
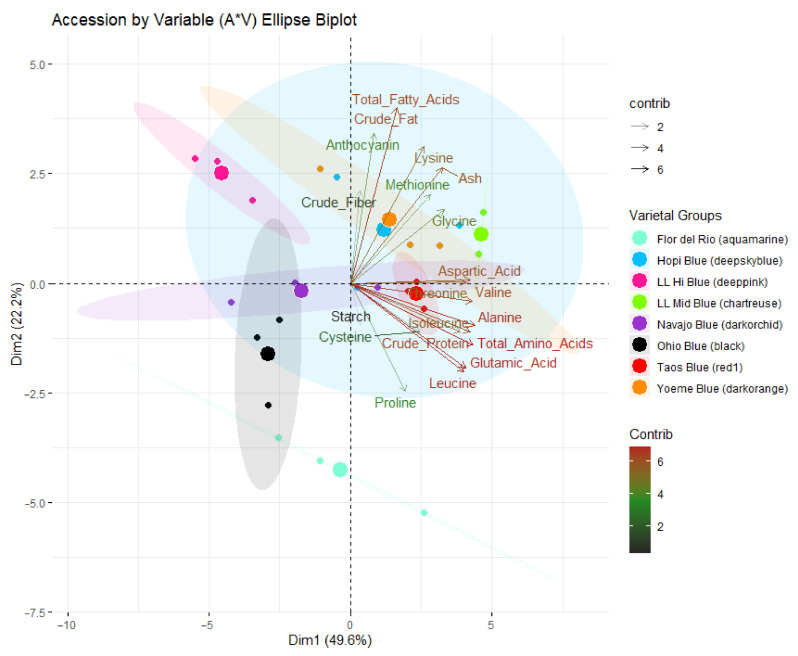
Accession by trait (A*T) biplot. Traits contributing to PC1 and PC2 are also assigned different gradient colors for a vector length 2, 4, and 6 with gray 15, Dodger blue, and firebrick, respectively. Small dots represent replicates of each accession, and the larger dot displays the center (mean) of the accession.

Four major principal components (PCs) with >1 eigenvalues were the most meaningful components and contributed to the majority of the variance (Table 6). The PC 1–4 explained a total of 85.26% cumulative variance with contributions of 49.62%, 22.20%, 6.98%, and 6.51% variance, respectively. The accession vs. trait biplot (A*T biplot) of PC1 and PC2 explained 71.77% of the variation (Figure 1) using the traits aspartic acid, threonine, glutamic acid, alanine, isoleucine, leucine, methionine, valine, lysine, total amino acids, protein, total fatty acids, crude fat, and anthocyanin (Table S3).

Traits contributing to PC1 and PC2 were assigned different gradient colors based on their contributions to the overall variation (Figure 1). Characters associated with starch and crude fiber contributed the least variation (vector length < 2), whereas anthocyanin, methionine, isoleucine, proline, cysteine, and glycine contributed moderate variation (vector length > 2 and ≤ 4). Moderate to the high variability of PC1 and PC2 was explained by total fatty acids, crude fat, lysine, ash, aspartic acid, valine, alanine, leucine, glutamic acid, threonine, and total amino acids, and crude protein (vector length of >4 and <6). Trait wise, alanine, valine, threonine, aspartic acid, total amino acids and total protein contributed strongly to PC1 variability (Table S3 and Figure 1), whereas anthocyanin, crude fat, total fatty acids contributed strongly to PC2 (Table S3 and Figure 1). Interestingly this suggests that PC1 aligns well with compounds containing nitrogen such as amino acids and protein (with the possible exceptions of lysine and proline), while PC2 seems to align well with non-nitrogen containing, carbon-rich compounds such as crude fat, total fatty acids, starch, anthocyanins, and crude fiber. The sulfur-containing amino acids cysteine and methionine are approximately equally aligned to both axes; however, PC3 aligns well with them (Table S3).

Blue maize accessions were distributed across all quadrants (Figure 1). Los Lunas Mid (ellipse with chartreuse color) was populated in quadrant 1 comprised of positive PC1 and PC2 (Figure 1), and characteristics of this quadrant are high crude fiber, anthocyanin crude fat, total fatty acids and amino acids (especially methionine, glycine and aspartic acid) and low starch. Los Lunas High (ellipse with deep pink color) was populated in quadrant 2, which comprised of a positive quadrant of PC2 and negative quadrant of PC1 and is characterized by low protein and starch whereas and high crude fiber, crude fat, and anthocyanin. Ohio blue (black color) was spread in quadrant 3 and characterized by high levels of starch within the accession, with generally low-fat levels (Figure 1). Hopi blue (ellipse with deep sky blue color) and Yoeme blue accessions (ellipse with dark orange color) were spread in quadrants 1, 2, and 4 (Figure 1). The variation within the Yoeme blue accession is strongly aligned with crude protein and total amino acids, while Hopi blue has many variations in each dimension. Navajo blue (ellipse with dark orchid color) was distributed across all four quadrants; however, the variation is clearly aligned with PC1, which indicates the little variation in fiber and starch, whereas high variation in certain amino acids. Flor del Rio (ellipse with aquamarine color) is distributed across quadrants 3 and 4 and characterized by high starch and low fiber and fat. Variation within the samples is well aligned with crude protein and total amino acids. Taos blue (ellipse with red1 color) is populated across quadrants 1 and 4 and has low within-accession variation, and is close to the average for most traits. The PCA helped to visualize the clustering of compounds, and to some extent, clusters of amino acids based on their biosynthetic pathways were seen. Amino acids from the aspartate (methionine, lysine, and aspartic acid), pyruvate (alanine, valine, and leucine), and glutamate (glutamic acid and proline) families were clustered together. Cysteine and glycine did not cluster well as a biosynthetic family.

#### 2.3.2. Correlation between Different Grain Compositional Traits and Correlation Network

**Figure 2:**
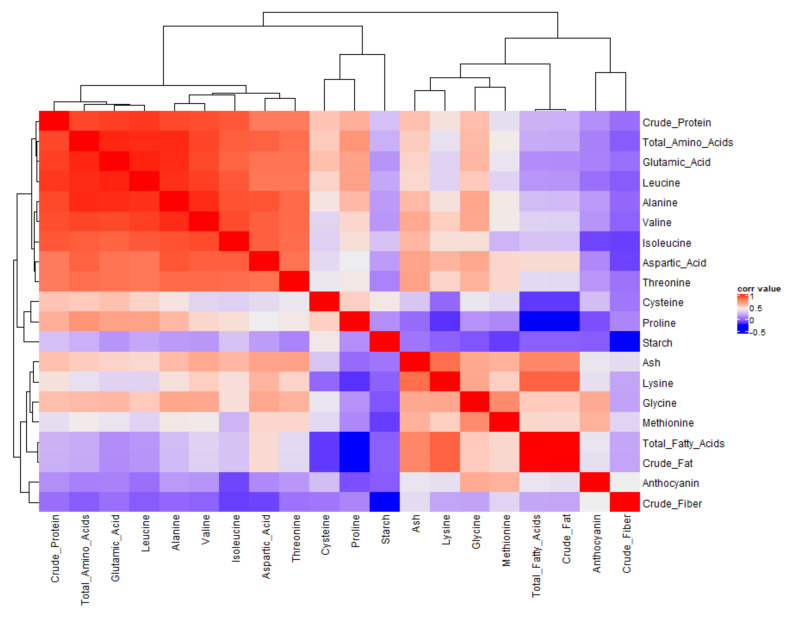
Correlation matrix heat map for different grain compositional traits.

**Figure 3:**
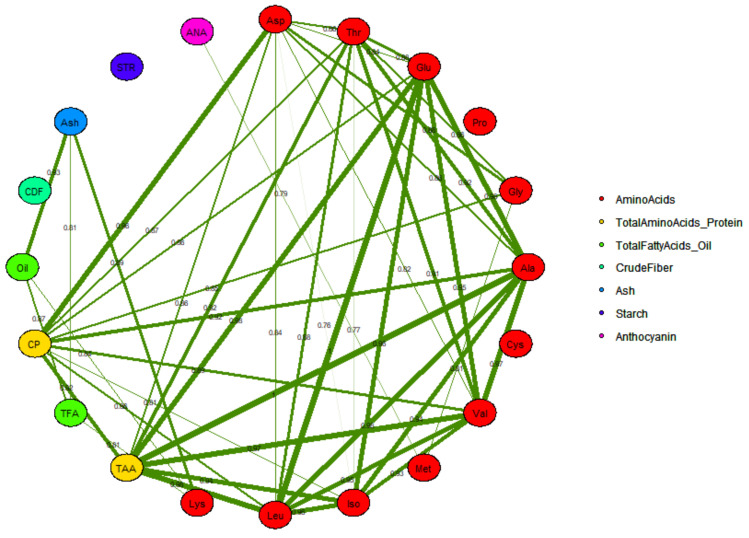
Blue maize grain compositional traits correlation network displaying the relationship between amino acids, total amino acids, total fatty acids, crude protein, oil, crude fiber, ash, starch, and anthocyanin. Abbreviations adapted for 20-grain compositional traits are shown in Table 1, and a total of 9 descriptor categories are used to display the relationship between amino acids, protein, oil, fiber, ash, starch, and anthocyanin. The number shown across each band represents the correlation coefficient between compositional traits. The width of each band represents the strength of correlation among traits, and the specific color of each ellipse represents the descriptor category assigned to that trait, as shown in the figure legend. The traits included in the correlation network are abbreviated as Asp: aspartic acid; Thr: threonine; Glu: glutamic acid; Pro: proline: Gly: glycine; Ala: alanine; Cys: cysteine; Val: valine; Met: methionine; Iso: isoleucine; Leu: leucine; Lys: lysine; TAA: total amino acids; TFA: total fatty acids; CP: crude protein; CDF: crude fiber; STR: starch; ANA: anthocyanin.

Correlations among grain compositional traits were investigated by a correlation matrix (Figure 2 and Table S4). Associations between traits were also illustrated by a correlation network (Figure 3). Strong correlations with an absolute value > 0.75 were included in the correlation network. In Figure 3, the width of each band represents correlation strength, whereas green colored bands illustrate positive correlations between descriptors. Most compositional traits were strongly correlated with another trait except for starch, anthocyanin, ash, crude fiber, and proline (Figure 3).

Some of the observed correlations can be explained by a mathematical relationship. Crude protein and total amino acids are essentially the same traits measured by two different methods. This may explain its strong correlation to total amino acids and crude protein. Crude fat and total fatty acids share a similar relationship. All of the amino acids are components of both crude protein and total amino acids, so some degree of correlation is expected. Crude fat and total fatty acids share a similar relationship. Most amino acids were strongly correlated with each other except for proline, and individual amino acids were also strongly correlated with total amino acids except proline, glycine, cysteine, and methionine (Figure 2 and Figure 3, Table S4). Glutamic acid has an important role in nitrogen assimilation and thus plays a role in the synthesis of all amino acids. This may explain its strong correlation to most amino acids and crude protein (Figure 2 and Figure 3, Table S4). Essential amino acids were not strongly correlated with protein with correlations of <0.60 (Figure 2 and Table S4). With regard to the relationship between anthocyanin and other compositional traits, no strong correlation was reported except for methionine (r = 0.79) and a moderate correlation with glycine (r = 0.69) and lysine (r = 0.62) (Figure 2 and Table S4). Similarly, starch had no strong or only a moderate positive correlation, with other components except leucine (r = 0.54); however, negative correlations were observed with total amino acids (r = −0.53), threonine (= −0.51), alanine (r = −0.53), leucine, glutamic acid (r = −0.55), and crude fiber (r = −0.49) (Figure 2 and Table S4). Oil and fatty acids were strongly correlated with ash and lysine only, whereas no significant moderate correlations were observed with other compositional traits (Figure 2 and Table S4). It is clearly noticeable that starch, anthocyanin, crude fiber, and proline were correlated with only a few or no other components and were most independent of other traits and may therefore be most valuable for classifying grain samples (Figure 2 and Figure 3, Table S4).

## 3. Discussion

Evaluated accessions were comprised of pop-flint and floury grain that displayed several colors. The varying degrees of hardness and pigmentation color may have been a cause of variation among accessions for total fatty acids, oil, protein, starch, and a degree of anthocyanin expression. In general, the ranges of protein, oil, starch, and anthocyanin are in agreement with the data reported in the literature for grain compositional analysis [13,14,38]. Among all amino acids evaluated, only methionine and lysine showed significant accession effects; however, the grain constituents of oil, fiber, ash, and anthocyanin also showed significant accession effects.

Multiple factors that could influence the performance of NIRS prediction calibration include the nature of the sample set and the accuracy of the analytical method [15,39]. Reproducibility of the reference analysis procedure being a limiting factor, as observed by Wehling et al. in a study of extractable starch [40]. In addition, the grain shape, size and uniformity of evaluated samples could also influence the results [41]. Past studies have shown that the use of full 700–2500 nm spectra reduces the influence of grain color on NIRS prediction ability as compared to the 400–2500 nm visible range [42]. We used 850 to 1048 nm spectral regions in our study. While our reference analysis was generally quite reproducible, the quality of our calibrations was likely limited by the small range of trait values in our data set. For example, the range of reference chemistry values in our data set was less than 1% of the sample mass.

Spielbauer et al. [21] and Meng et al. [13] reported PLS as best prediction method; however, Meng et al. have suggested that a PLS model be selected since it was suitable for both whole grain and ground samples. Many other studies have used this method [8,20,43]. We used PCR and PLS calibration models to develop calibrations to predict 20 constituents in the pigmented maize samples analyzed in this study. These calibrations were validated with an independent subset of the data. Based on RMSEP and RPD, the usefulness of the calibration models is poor, and the results can probably be greatly improved by adding samples with a greater range in trait values to the sample set.

Principal component analysis was utilized to ascertain divergence between populations, and it further supported the existing variability reported in ANOVA. Eigenvector-derived PCs indicate that anthocyanin, lysine, total amino acids, total fatty acids, and protein were the most discriminative traits and can be used to characterize the pigmented grain compositional diversity. Our findings are in alignment with an earlier report of the same experimental material that was evaluated for agronomic and compositional trait variability [36]. The experimental material was comprised of different grain colors, architecture, and size varying from blue to reddish-purple in color and floury to pop-flint grain texture. Measured variation was likely confounded with other traits, which may have caused overlapping of different accessions, and observed variability for grain compositional traits is likely associated with variation caused by year and location [28]. Meng et al. [13] made similar observations and highlighted the influence of grain compositional traits in biochemical variability and their potential role in germplasm improvement.

A correlation network was constructed using highly correlated traits (r = >0.75), and it further validated the relationship observed among compositional traits and exhibited relatedness within grain quality traits. Many of the observed correlations can be explained by the common biosynthetic origins of the compounds examined. For example, alanine, leucine, isoleucine, and valine are in the pyruvate biosynthetic family [44]. Similarly, anthocyanins are synthesized from malonyl CoA, a compound in the lipid biosynthetic pathway [45,46], explaining the positive correlations between anthocyanins, crude fat, and total fatty acids. Some compounds with similar biosynthetic origins do not share strong correlations. For example, fat, crude fiber and starch are both made from glucose, yet they are negatively correlated (Figure 3), and Lane et al. [47] also reported a very poor correlation between starch and fat (r = <0.17). This negative correlation is likely due to the very different functional roles played by the compounds. Starch is an energy storage compound, while most of the crude fiber has a structural role in cell walls [48]. Similarly, cysteine and methionine share relatively poor correlations with most other protein-related traits (Figure 2 and Table S4). While both are components of proteins, they both are sulfur amino acids with additional roles in metabolism. Methionine functions in one-carbon metabolism as S-adenosyl methionine, and cysteine functions in redox homeostasis as a component of glutathione [49].

Our current calibrations are based on the analysis of whole grains. Considering previous comparative assessments of whole-grain and ground grain [6,13,17,20], it would be worthwhile to investigate further the effect of grinding on calibration accuracy, robustness, and reliability. However, NIRS with whole grains is 3–4 times faster than with the ground grains, even without considering the added time required for grinding [6]. The speed of whole-grain NIRS could facilitate the expedient selection between harvest and planting of the next generation, and the additional speed may be worth some loss in quality of prediction in some situations.

## 4. Materials and Methods

### 4.1. Experimental Design and Plant Material

#### 4.1.1. Experimental Design

Grain samples comprised of bulked grain from open-pollinated hand-harvested ears from the field trials conducted during 2013 and 2014 at several New Mexico locations. In 2013, trails were planted in Las Cruces, Los Lunas, Farmington, and Alcalde locations, and in 2014, only Los Lunas and Alcalde were used. At each testing environment, all trials were conducted with three replications in a randomized complete block design (RCBD).

#### 4.1.2. Plant Material

Grain samples of eight different landraces of pigmented maize from the southwestern USA were used in this study. These landrace accessions were primarily floury endosperm types with diverse color phenotypes, including blue, purple, red, and reddish-purple. A midwestern corn belt dent type (Ohio blue) was used as a control for varietal comparison. Several of the landrace accessions also included some dent and small pop flint grain types. At the end of the growing season, all accessions were allowed to air dry in the field before being hand-harvested and harvested samples shelled manually to avoid contamination. Samples were stored in a dark, cold room and allowed to equilibrate to room conditions for several days before analysis.

#### 4.1.3. Sample Preparation

Whole grain near-infrared reflectance spectroscopy of grain samples was done on a per-plot basis using the bulked grain of all ears in the plot. Near-infrared reflectance spectra for the whole grains of pigmented maize were scanned using a Foss 1241-grain analyzer. All NIR measurements were reported on a dry weight basis. The set of 143 samples was used to develop calibration equations for protein, oil, starch, total fiber, ash, crude fat, ash, fiber, and a group of amino acids, including cysteine, methionine, lysine, aspartic acid, threonine, glutamic acid, proline, glycine, alanine, valine, isoleucine, and leucine.

### 4.2. NIR Spectral Data Collection and Pretreatments

All spectral data were obtained using a Foss 1241-grain analyzer infrared spectrophotometer (Foss NIRSystem, Silver Spring, MD, USA). The reflectance spectrum representing each sample was an average of 5 independent subsample spectra measured and was collected at 1-nm intervals between 850 to 1048 nm.

### 4.3. Wet Chemistry for Grain Constituent Analysis

After NIR scanning and data collection, all samples were milled for reference analysis using a laboratory mill with a 0.5 mm sieve (Glenn Mills, Clifton, NJ, USA) and grain compositional constituents were analyzed on a dry matter basis. All samples in the calibration set were tested for grain composition constituents using wet-chemistry at the Experiment Station Chemical Laboratory (ESCL) at the University of Missouri, Columbia, MO, USA. Compositional constituent included in reference analysis were analyzed by standard approved reference analysis procedures: protein, Kjeldahl (AOAC Official Method 990.03) [50]; oil, ether extraction (AOAC Official method 920.29 (A) [51]; starch (Amer. Cereal Chemists, approved methods, no. 76–13) [52]; total fatty acids (AOCS Official method Ca 5b-71) [53]; fiber (AOAC Official method 978.10) [54]; anthocyanin [55], and total amino acids including essential amino acids of cysteine, methionine and lysine (AOAC International method 994.12 methods) [56].

### 4.4. Data Preprocessing and Construction of Calibration Equation

#### 4.4.1. Data Preprocessing

Prior to developing a chemometrics model for calibration data transformations unrelated to compositional constituents and unprocessed spectra’s physical metrics were corrected by preprocessing tools. Multiplicative signal correction (MSC) and standard normal variate tools were used in Unscrambler^®^ 9.8 software (Camo Software, Oslo, Norway) using the default setting. Data smoothing was conducted using zero and first derivative (D0 and D1) to reduce the scatter effect, baseline shift, and path length differences [13,57].

#### 4.4.2. Construction of Calibration Equations

Calibration equations to predict compositional constituents on a dry weight basis from each set of spectral data were developed by different chemometric models, including principal component regression (PCR) and partial least square (PLS) provided in the multi-variate analysis software package of Unscrambler^®^. The PCR and PLS prediction methods were evaluated to determine which could better predict the compositional constituents of the grains. In each model, the spectral data were used as descriptor data, and wet chemistry compositional data were used as response data to predict the grain compositional constituents. Seven significant factors for PCR and PLS were determined based on cross-validation. In PLS, a linear combination of spectral wavelength and wet lab reference data are used to develop the calibration model [18]. Calibration or validation data sets were determined based on the sample sorting by compositional constituents, and among all samples, every third sample was included in the validation set while the rest of the samples comprised the calibration set.

### 4.5. Statistical Analysis

NIR calibration equations were developed for grain proximates as well as essential and conditionally essential amino acids and total fiber content using Unscrambler^®^ 9.8 software program. Correlations between NIR spectra-based predictions and wet chemistry data across (R^2^_val_) compositional constituent values, root mean square error of validation set (RMSEP) and the ratio of the SEP to the standard deviation of measured compositional constituents (RPD). RMSEP estimates were calculated to determine the differences between NIRS predictions and reference analysis results. SAS software version 9.3 was used to do descriptive statistics and combined analysis of variance (ANOVA) of wet lab data (SAS Institute, Cary, NC, USA). Principal component analysis was used to understand the variation between accessions. Eigenvalue, eigenvector, percent variance of different principal components and accession by trait biplot were estimated by *ggplot2* [58], *missMDA* [59], *FactoMineR* [60], and *Factoextra* [61] R packages. The correlation coefficient matrix and the correlation network were constructed to understand how different compositional traits contribute to grain biochemical diversity. The correlation matrix and correlation network were constructed using *ComplexHeatmap* [62] and *qgraph* [63], respectively.

## 5. Conclusions

Results reveal the challenges of using NIRS to predict many grain constituents. Calibrations for most components were poor and are not recommended to use except for methionine and glycine. A plausible reason for poor calibrations is that very little variation was observed included in the experiment. The relationship among grain components studied by PCA demonstrated that PCs tend to align with elements contained in the compounds examined: PC1 with nitrogen (amino acids), PC2 with carbon (starch, anthocyanin, fiber, and fat), and PC3 with the sulfur compounds (cysteine and methionine). Correlation analysis showed that many relationships among traits reflect either biosynthetic origin as observed for amino acids from the pyruvate biosynthetic family (alanine, leucine, and valine) or anthocyanins, crude fat and fatty acids, which are all derived from malonyl CoA. Other correlations may be derived from functional relationships as seen with crude fiber and starch, which have similar biosynthetic origins, but different functional roles.

## Figures and Tables

**Table 1:** Descriptive statistics of wet lab analyzed grain compositional traits. Amino acids (Asp, Thr, Glu, Pro, Gly, Ala, Cys, Val, Met, Iso, Leu, Lys), TAA, TFA, CP, Oil, CDF, Ash, and STR were measured in % while anthocyanin was measured in mg/100 g.

Trait	Code	N	Min	Mean	Max	SD	CV (%)
Aspartic acid	Asp	143	0.69	0.71	0.73	0.09	8.07
Threonine	Thr	143	0.33	0.34	0.35	0.04	8.21
Glutamic acid	Glu	143	1.81	1.93	1.99	0.27	9.97
Proline	Pro	143	0.88	0.93	0.97	0.13	13.13
Glycine	Gly	143	0.38	0.39	0.40	0.04	5.90
Alanine	Ala	143	0.79	0.83	0.86	0.12	9.56
Cysteine	Cys	143	0.20	0.21	0.22	0.02	6.47
Valine	Val	143	0.49	0.50	0.52	0.06	8.76
Methionine	Met	143	0.25	0.26	0.28	0.04	11.53
Isoleucine	Iso	143	0.34	0.36	0.37	0.06	9.07
Leucine	Leu	143	1.29	1.39	1.44	0.22	11.17
Lysine	Lys	143	0.31	0.32	0.33	0.03	5.79
Total amino acids	TAA	143	7.81	8.18	8.44	1.02	8.90
Total fatty acids	TFA	143	3.76	5.48	6.26	1.49	20.69
Protein	CP	143	10.35	10.86	11.29	1.21	8.15
Oil	Oil	143	3.89	5.68	6.49	1.54	20.97
Crude fiber	CDF	143	1.61	1.68	1.81	0.18	8.62
Ash	Ash	143	1.41	1.52	1.64	0.14	7.34
Starch	STR	143	55.60	56.47	57.70	2.83	4.70
Anthocyanin	ANA	143	16.67	46.23	58.24	20.57	23.83

**Table 2:** Mean squares and ANOVA of grain compositional traits evaluated across locations and years. *, **, *** significant at P < 0.05, 0.01, and 0.001, respectively.

Trait	Accession	Location	Year	Interaction			
				A × L	A × Y	L × Y	A × L × Y
Aspartic acid	1.19	20.15 ***	65.48 ***	1.08	2.31 *	0.03	0.93
Threonine	1.43	8.41 ***	75.71 ***	1.32	2.42 *	9.27 **	0.51
Glutamic acid	1.66	9.88 ***	65.14 ***	1.28	1.96	0.27	0.90
Proline	0.92	3.50 *	11.30 **	0.92	0.47	0.01	0.42
Glycine	1.67	16.37 ***	117.43 ***	0.81	1.09	0.78	0.37
Alanine	1.62	12.95 ***	72.20 ***	1.33	1.96	0.01	0.96
Cysteine	1.94	28.65 ***	90.36 ***	1.60	0.87	3.73	0.64
Valine	1.05	4.54 **	55.78 ***	0.95	1.86	6.59	1.09
Methionine	3.00 **	11.96 ***	81.99 ***	0.95	1.09	13.52 ***	1.01
Isoleucine	1.21	8.25 ***	107.05 ***	1.24	2.06	8.32 ***	1.29
Leucine	1.59	12.64 ***	60.73 ***	1.33	1.83	0.18	1.15
Lysine	4.00 ***	17.85 ***	14.60 ***	0.48	1.75	0.11	0.47
Total amino acids	1.20	9.37 ***	66.70 ***	1.18	1.76	0.24	0.90
Total fatty acids	9.29 ***	2.01	3.19	1.17	1.25	3.37	2.13 *
Protein	1.62	15.33 ***	44.39 ***	0.94	1.74	1.00	0.83
Oil	9.29 ***	2.00	3.15	1.16	1.25	3.34	2.14 *
Crude fiber	4.03 ***	4.35 **	16.33 ***	1.08	0.36	17.26 ***	0.70
Ash	7.48 ***	6.07 ***	2.03	0.82	0.86	0.00	1.31
Starch	1.69	5.19 **	4.43 *	0.73	1.30	0.09	0.91
Anthocyanin	18.04 ***	18.88 ***	14.57 ***	2.70 ***	2.97 **	16.17 ***	3.70 **

**Table 3:** Standard errors of near-infrared reflectance (NIR) predicted values and reference analysis of various grain constituents obtained from blue maize grain samples. Root mean squares of calibration set (RMSEP) calculated for principal component regression (PCR) and partial least square (PLS) calibration models.

Constituent	NIR	LAB	RMSEP	
			PCR	PLS
Protein (%)	0.123	0.102	1.11	1.05
Oil (%)	0.107	0.129	1.44	1.43
Starch (%)	0.170	0.238	2.81	2.80
Lysine (%)	0.009	0.002	0.02	0.02
Methionine (%)	0.007	0.004	0.33	0.03
Cysteine (%)	0.011	0.002	0.28	0.02

**Table 4:** PCR and PLS model results of calibration and validation for twenty different constituents (0th derivative). Coefficient of determination (R^2^) of actual (R^2^_cal_) and predicted (R^2^_val_) constituent values. Ratio of performance to the standard deviation (RPD).

	PCR						PLS					
	Calibration			Validation			Calibration			Validation		
Trait	R^2^_cv_	RMSEC	R^2^_cal_	R^2^_val_	RMSEP	RPD	R^2^_cv_	RMSEC	R^2^_cal_	R^2^_val_	RMSEP	RPD
Asp	0.37	0.08	0.19	0.14	0.08	0.93	0.49	0.07	0.30	0.25	0.07	0.81
Thr	0.31	0.04	0.17	0.10	0.04	0.91	0.44	0.04	0.26	0.20	0.04	0.91
Glu	0.34	0.25	0.25	0.25	0.12	0.44	0.48	0.23	0.28	0.23	0.24	0.89
Pro	0.04	0.13	0.0001	NA	0.13	1.03	0.22	0.13	0.002	NA	0.13	1.03
Gly	0.51	0.03	0.31	0.27	0.03	0.83	0.59	0.03	0.38	0.35	0.03	0.83
Ala	0.38	0.19	0.10	0.15	0.11	0.95	0.49	0.10	0.30	0.25	0.10	0.86
Cys	0.47	0.02	0.28	0.23	0.02	0.83	0.49	0.02	0.29	0.24	0.02	0.83
Val	0.22	0.10	0.06	0.04	0.06	0.98	0.43	0.05	0.24	0.18	0.05	0.82
Met	0.51	0.04	0.33	0.27	0.04	0.93	0.60	0.03	0.40	0.36	0.03	0.70
Iso	0.39	0.05	0.19	0.15	0.05	0.91	0.54	0.05	0.34	0.29	0.05	0.91
Leu	0.33	0.20	0.15	0.12	0.21	0.95	0.45	0.19	0.27	0.21	0.20	0.90
Lys	0.21	0.02	0.08	0.05	0.02	0.80	0.30	0.02	0.16	0.09	0.02	0.80
TAA	0.33	0.93	0.18	0.12	0.97	0.95	0.49	0.86	0.29	0.25	0.89	0.87
TFA	0.31	1.39	0.12	0.11	1.41	0.95	0.32	1.38	0.13	0.12	1.40	0.94
CP	0.31	1.11	0.15	0.10	1.15	0.95	0.45	1.05	0.24	0.20	1.09	0.17
Oil	0.30	1.44	0.12	0.10	1.46	0.95	0.33	1.43	0.13	0.11	1.45	0.94
CDF	0.20	0.17	0.09	0.04	0.18	0.99	0.30	0.14	0.17	0.10	0.17	0.94
Ash	0.18	0.13	0.05	0.04	0.13	0.96	0.23	0.13	0.08	0.06	0.13	0.96
STR	0.45	2.81	0.0001	NA	2.86	1.01	0.16	2.8	0.01	NA	2.86	1.01
ANA	0.25	19.2	0.13	0.07	19.9	0.97	0.37	18.9	0.15	0.10	19.6	0.95

**Table 5:** PCR and PLS model results of calibration and validation for twenty different constituents (1st Derivative) of blue maize grain samples. Coefficient of determination (R^2^) of actual (R^2^_cal_) and predicted (R^2^_val_) constituent values. Ratio of performance to the standard deviation (RPD).

	PCR						PLS					
	Calibration			Validation			Calibration			Validation		
Trait	R^2^_cv_	RMSEC	R^2^_cal_	R^2^_val_	RMSEP	RPD	R^2^_cv_	RMSEC	R^2^_cal_	R^2^_val_	RMSEP	RPD
Asp	0.37	0.08	0.19	0.14	0.08	0.93	0.52	0.07	0.30	0.27	0.07	0.81
Thr	0.31	0.04	0.14	0.10	0.04	0.91	0.47	0.04	0.27	0.23	0.04	0.91
Glu	0.34	0.25	0.17	0.25	0.12	0.44	0.51	0.23	0.29	0.26	0.23	0.85
Pro	0.04	0.13	0.001	NA	0.13	1.03	0.10	0.12	0.3	NA	0.13	1.03
Gly	0.51	0.03	0.30	0.27	0.03	0.83	0.59	0.03	0.39	0.36	0.03	0.83
Ala	0.38	0.10	0.19	0.15	0.11	0.95	0.54	0.10	0.31	0.30	0.10	0.86
Cys	0.47	0.02	0.28	0.23	0.02	0.83	0.52	0.02	0.33	0.27	0.02	0.83
Val	0.22	0.10	0.06	0.04	0.06	0.98	0.45	0.05	0.25	0.24	0.05	0.82
Met	0.51	0.03	0.33	0.27	0.04	0.93	0.36	0.03	0.40	0.36	0.03	0.70
Iso	0.38	0.05	0.19	0.15	0.05	0.91	0.56	0.04	0.37	0.32	0.05	0.91
Leu	0.33	0.20	0.15	0.12	0.21	0.95	0.47	0.19	0.27	0.20	0.22	1.00
Lys	0.21	0.02	0.10	0.05	0.02	0.80	0.31	0.02	0.15	0.10	0.02	0.80
TAA	0.33	0.92	0.18	0.12	0.97	0.95	0.51	0.85	0.30	0.27	0.88	0.86
TFA	0.31	1.39	0.12	0.11	1.41	0.95	0.30	1.39	0.12	0.10	1.41	0.95
CP	0.31	1.11	0.15	0.10	1.15	0.95	0.45	1.05	0.25	0.22	1.08	0.89
Oil	0.30	1.44	0.12	0.10	1.46	0.95	0.30	1.44	0.12	0.11	1.46	0.95
CDF	0.20	0.17	0.09	0.04	0.18	0.99	0.31	0.17	0.14	0.10	0.17	0.94
Ash	0.18	0.13	0.05	0.04	0.13	0.96	0.19	0.13	0.06	0.04	0.13	0.96
STR	0.45	2.81	0.001	NA	2.86	1.01	0.23	2.77	0.03	NA	2.90	1.02
ANA	0.25	19.2	0.13	0.07	19.9	0.97	0.27	18.9	0.15	0.08	19.9	0.97

**Table 6:** Eigenvalue, variance contribution (%), and total cumulative variance (%) of principal components.

Principal Components	Eigenvalue	Variance (%)	Cumulative Variance (%)
1	9.92	49.62	49.62
2	4.43	22.20	71.77
3	1.40	6.98	78.75
4	1.30	6.51	85.26
5	0.90	4.48	89.74
6	0.67	3.32	93.06
7	0.38	1.92	94.98
8	0.30	1.52	96.50
9	0.20	1.09	97.59
10	0.15	0.73	98.32

## Supplementary Materials

The following are available online at https://www.mdpi.com/2223-7747/9/12/1775/s1: Table S1A: Calibration parameters of PCR and PLS for 0th derivative; Table S1B: Calibration parameters of PCR and PLS for the first derivative; Table S2A: Calibration equations (B-coefficients) for grain constituent estimated with 0th derivative; Table S2B: Calibration equations (B-coefficients) for grain constituent estimated with the first derivative; Table S3: Grain compositional trait contribution (PC feature), correlation coefficient (PC_R2), and eigenvector (Eigen_Vector) for principal components 1, 2, and 3; Table S4: Correlation matrix of grain compositional traits; the upper triangle represents correlation (R2), whereas the lower triangle represents p values between compositional traits.
