# Supplementary material for: Compositional Analyses Reveal Relationships among Components of Blue Maize Grains
Source: Plants (Basel). 2020 Dec 14;9(12):1775. doi: 10.3390/plants9121775 (PMC7765092; doi:10.3390/plants9121775)
Supplement: Supplementary file 1 [file plants-09-01775-s001.zip › Supplementary Materials/S3 Table. PCA variable contribution.docx]

**Table S3. Kernel compositional trait contribution (PC_Feature), correlation coefficient (PC_R^2^), and eigen vector (Eigen_Vector) for principle components 1, 2, and 3.**

| **Trait** | **Features** | | |  | **Correlation coefficient (R^2^)** | | |  | **Eigen vector** | | |  |
| --- | --- | --- | --- | --- | --- | --- | --- | --- | --- | --- | --- | --- |
|  | **PC1** | **PC2** | **PC3** |  | **PC1** | **PC2** | **PC3** |  | **1** | **2** | **3** |  |
| **Aspartic Acid** | 8.56 | 0.01 | 3.05 |  | 0.92 | 0.02 | -0.21 |  | -0.28 | 0.00 | 0.16 |  |
| **Threonine** | 7.95 | 0.001 | 0.56 |  | 0.89 | 0.01 | -0.09 |  | -0.27 | 0.03 | 0.03 |  |
| **Glutamic Acid** | 7.95 | 4.02 | 0.05 |  | 0.89 | -0.42 | 0.02 |  | -0.28 | 0.18 | -0.07 |  |
| **Proline** | 1.81 | 6.51 | 4.85 |  | 0.42 | -0.54 | 0.26 |  | -0.16 | 0.30 | -0.24 |  |
| **Glycine** | 5.22 | 3.05 | 2.10 |  | 0.72 | 0.37 | 0.17 |  | -0.23 | -0.19 | -0.18 |  |
| **Valine** | 8.87 | 0.18 | 1.23 |  | 0.94 | -0.09 | -0.13 |  | -0.29 | 0.07 | 0.07 |  |
| **Cysteine** | 2.86 | 1.31 | 28.39 |  | 0.53 | -0.24 | 0.63 |  | -0.15 | 0.16 | -0.33 |  |
| **Alanine** | 9.24 | 1.01 | 0.22 |  | 0.96 | -0.21 | -0.06 |  | -0.29 | 0.11 | 0.00 |  |
| **Methionine** | 3.81 | 4.41 | 14.37 |  | 0.61 | 0.44 | 0.45 |  | -0.18 | -0.24 | -0.30 |  |
| **Isoleucine** | 7.17 | 1.32 | 7.43 |  | 0.84 | -0.24 | -0.32 |  | -0.27 | 0.12 | 0.22 |  |
| **Leucine** | 7.71 | 4.40 | 0.16 |  | 0.87 | -0.44 | -0.05 |  | -0.28 | 0.19 | 0.00 |  |
| **Lysine** | 3.23 | 10.40 | 4.47 |  | 0.57 | 0.68 | -0.25 |  | -0.19 | -0.34 | 0.19 |  |
| **Total Fatty Acids** | 1.28 | 17.10 | 3.31 |  | 0.36 | 0.87 | -0.21 |  | -0.14 | -0.40 | 0.22 |  |
| **Total Amino Acids** | 9.03 | 2.14 | 0.05 |  | 0.95 | -0.31 | 0.03 |  | -0.29 | 0.16 | -0.02 |  |
| **Protein** | 8.54 | 1.32 | 0.26 |  | 0.92 | -0.24 | 0.06 |  | -0.28 | 0.13 | 0.00 |  |
| **Fiber** | 0.05 | 4.74 | 16.60 |  | 0.07 | 0.46 | 0.48 |  | -0.03 | -0.21 | -0.49 |  |
| **Ash** | 5.10 | 7.43 | 0.001 |  | 0.71 | 0.57 | 0.00 |  | -0.23 | -0.26 | 0.05 |  |
| **Crude Fat** | 1.27 | 17.10 | 3.30 |  | 0.36 | 0.87 | -0.21 |  | -0.14 | -0.40 | 0.23 |  |
| **Starch** | 0.07 | 0.99 | 0.04 |  | 0.08 | -0.21 | 0.02 |  | -0.06 | 0.15 | 0.26 |  |
| **Anthocyanin** | 0.34 | 12.50 | 9.56 |  | 0.18 | 0.75 | 0.37 |  | -0.09 | -0.27 | -0.42 |  |
